# The evolution of opsin genes in five species of mirid bugs: duplication of long-wavelength opsins and loss of blue-sensitive opsins

**DOI:** 10.1186/s12862-021-01799-5

**Published:** 2021-04-26

**Authors:** Pengjun Xu, Bin Lu, Jiangtao Chao, Robert Holdbrook, Gemei Liang, Yanhui Lu

**Affiliations:** 1 Tobacco Research Institute, Chinese Academy of Agricultural Sciences, Qingdao, 266101 People’s Republic of China; 2 State Key Laboratory for Biology of Plant Diseases and Insect Pests, Institute of Plant Protection, Chinese Academy of Agricultural Sciences (IPP-CAAS), Beijing, 100193 People’s Republic of China; 3 Department of Herpetology, Chengdu Institute of Biology, Chinese Academy of Sciences, Chengdu, Sichuan 610041 People’s Republic of China; 4 Lancaster Environment Centre, Lancaster University, Lancaster, LA1 4YQ UK

**Keywords:** Opsin, Miridae, Evolution, Duplication, Expression

## Abstract

**Background:**

Color vision and phototactic behavior based on opsins are important for the fitness of insects because of their roles in foraging and mate choice. Related topics, including the duplication and loss of opsin genes, have been well investigated in insect orders such as Coleoptera, Lepidoptera, Hymenoptera, Odonata and Orthoptera, and the findings have been used to develop pest management strategies involving light trapping. Mirid bugs of Hemiptera, which are pests that cause heavy economic losses, show capacity for color discrimination and phototaxis. However, the opsins in mirid bugs remain uncharacterized. Herein, we examined five species to investigate the evolution of opsins in the family Miridae.

**Results:**

Using RNA-seq, we identified several contigs showing high identity with opsins, including four contigs in *Apolygus lucorum* and three contigs each in *Adelphocoris suturalis*, *Adelphocoris fasciaticollis*, *Adelphocoris lineolatus* and *Nesidiocoris tenuis*. Phylogenetic analyses indicated that one of these genes clustered with ultraviolet-sensitive (UV) opsins and that the others clustered with long-wavelength (LW) opsins, suggesting that duplication of LW opsins and loss of blue light-sensitive (B) opsins occurred in mirid bugs. The existence of introns in the LW opsins of mirid bugs suggested that the duplication events were DNA based. Both LW1 and LW2 opsins of mirid bugs were found to be under strong purifying selection. The LW1 opsins were significantly more highly expressed than the LW2 and UV opsins.

**Conclusions:**

We identified the opsins of mirid bugs using five selected mirid species as a representative sample. Phylogenetic analyses clustered one of the genes with UV opsins and the others with LW opsins, suggesting the occurrence of LW opsin duplication and B opsin loss during the evolution of mirid bugs. Intron detection suggested that the identified duplication event was DNA based. The evidence of strong purifying selection and the relatively high expression levels suggested that these opsins exhibit fundamental functions in mirid bugs.

**Supplementary Information:**

The online version contains supplementary material available at 10.1186/s12862-021-01799-5.

## Background

The family *Miridae* (Hemiptera: Heteroptera), members of which are also known as “plant bugs”, is one of the most diverse families of insects, including approximately 11,020 species in more than 1200 genera [1, 2]. The compound eyes of these insects are usually large, and ocelli are absent, except in species from the subfamily Isometopinae [1, 2]. According to their feeding habits and host ranges, mirid bugs are divided into two main groups: phytozoophages (herbivores that complement their diets with prey; some species are important agricultural pests, such as *Apolygus lucorum* and *Adelphocoris suturalis*) and zoophytophages (predators that occasionally feed on plant resources and are considered natural enemies useful for pest management, such as *Nesidiocoris tenuis* and *Macrolophus pygmaeus*) [2–5]. Recently, several mirid bugs (e.g., *Ap. lucorum*, *Ad. suturalis*) have attracted much attention because they feed on more than 100 plant species and cause significant economic losses [4–7]. Given their color vision and positively phototactic behavior, color and light traps are used to monitor and manage these nocturnal pests [8–10]. A previous study has revealed that adults of *Ap. lucorum* are significantly more attracted by green (515–518 nm) LEDs than by red (587–590 nm) and yellow (615–618 nm) LEDs [10]. Consistent with this finding, separate studies have indicated that green traps are most attractive to *Ap. lucorum* [11, 12]. Opsins play central roles in the color vision and phototaxis of insects [13–15]. However, the details of opsin evolution in mirid bugs remains unclear.

Opsins are G-protein-coupled receptors characterized by seven transmembrane-domain structures, which determine the spectral sensitivity of the photopigment, and a light-sensitive vitamin A-derived chromophore that is characterized by a lysine residue in the seventh helix [16]. Physiological and molecular phylogenetic analyses have revealed that ancient insects possessed trichromatic vision involving three subfamilies of visual opsins: long-wavelength-sensitive (LW) opsins (> 500 nm), blue light-sensitive (B) opsins (400–500 nm) and ultraviolet-sensitive (UV) opsins (325–400 nm) [13, 17]. Color vision based on opsin photoreceptor molecules plays an important role in survival-related behaviors of insects (e.g., foraging, mating choice) [18–20], and insects have evolved diverse types of color vision via duplication and loss of opsin genes. For example, *Drosophila* has evolved a fourth subfamily composed of blue-green-sensitive (BG) opsins (approximately 480 nm) [13, 17, 21, 22], dragonflies have undergone duplication of opsins [23, 24], and beetles have lost B opsins [25–27]. Based on studies of opsin molecular evolution, trichromatic vision is considered an ancestral trait determined by opsin loss or gain in insects [13, 17]. In addition to color vision, opsins play important roles in the phototactic behavior of insects that are used worldwide for integrated pest management [14, 15]. Over the last several decades, the opsins of insects have been well studied in Lepidoptera, Coleoptera, Hymenoptera, Odonata and Orthoptera [23, 24, 26–31]. For example, related studies have revealed that duplication and mutation of opsin genes have expanded spectral diversity in lepidopteran insects to increase their capacity for color vision [32, 33], and coleopteran insects have been used as models for investigation into how trichromacy can be achieved in the absence of B opsins [25–27]. However, no opsins have been reported in true bugs (Hemiptera: Heteroptera).

To better understand the molecular evolution of opsins in mirid bugs, we performed transcriptomic analyses on the opsin genes of five species from the two groups, including four phytozoophagous species (*Ap. lucorum, Ad. suturalis*, *Ad. fasciaticollis* and *Ad. lineolatus* [6, 34, 35]) and one zoophytophagous species (*N. tenuis* [2, 3]). We identified four opsin-like contigs in *Ap. lucorum* and three in each of the other mirid bug species. Subsequent phylogenetic analyses suggested that duplication of LW opsins and loss of B opsins have occurred in mirid bugs. After gene duplication, genes tend to be subject to different levels of selection pressure, measured as the ratio between synonymous and nonsynonymous substitutions (*dn/ds*). The duplication event was found to be DNA based, and both LW1 and LW2 were found to be under strong purifying selection. These results account for the high expression levels of opsins and suggest the evolutionary mechanism of opsins in mirid bugs: functional LW opsins were obtained via DNA-based duplication, and B opsins were lost. These results could help us to develop novel management strategies for controlling mirid bugs by enhancing understanding of opsin-based color vision and phototaxis.

## Results

### Identification of opsin genes in five species of mirid bugs through transcriptome analyses

We obtained 4.8 gigabases (Gb) of clean data for *Ap. lucorum*. An overview of the sequencing and assembly data is provided. The RNA-seq data were submitted to the Sequence Read Archive (SRA) database (accession number: SRR6371236). Functional annotation of 22,771 unigenes (31.99%) was performed using the BLAST NR database with an E-value cutoff of 1e−5. Using the resulting data and RNA-seq data we obtained previously [35], we identified visual opsins in the five species of mirid bugs (Additional file 1: Table S1). According to these reference sequences, we designed primers and sequenced these contigs again via Sanger sequencing (Additional file 1: Table S2).

### Sequence alignment and phylogenetic analyses

**Fig. 1:**
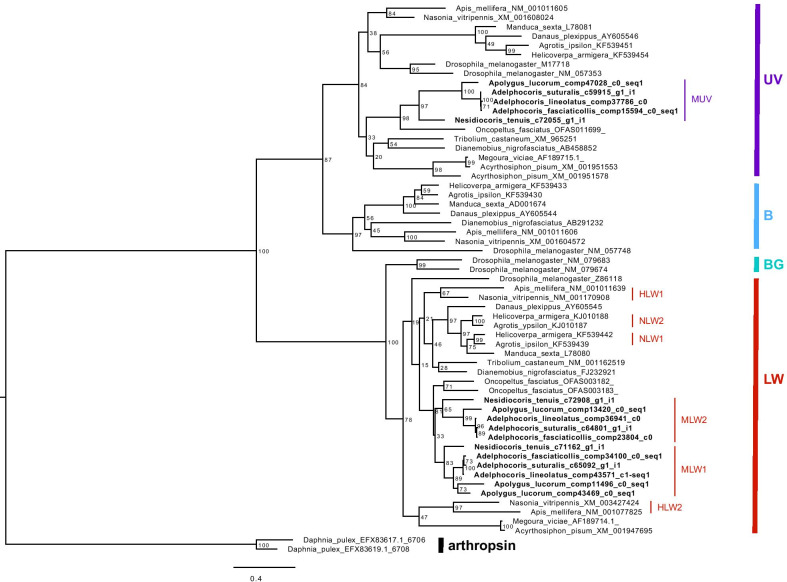
Phylogenetic reconstruction of opsins in insects and duplication of LW opsins in mirid bugs. The bootstrap values are shown on the nodes. *B* blue-sensitive opsin, *UV* ultraviolet-sensitive opsin, *BG* blue-green-sensitive opsin, *LW* long-wavelength-sensitive opsin. *HLW* LW opsin in species from Hymenoptera, *NLW* LW opsin from noctuid species in Lepidoptera, *MLW* LW opsin in species from *Miridae* in Hemiptera, *MUV* UV opsin in species from *Miridae* in Hemiptera

**Fig. 2:**
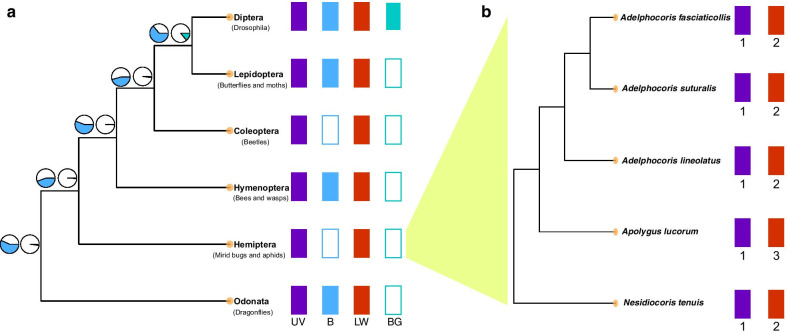
Opsin-based evolution of visual structure in insects. **a** Gains and losses of opsin genes in insect orders and ancestral state reconstruction. The colors represent the opsin types. The empty and color-filled rectangles represent losses and gains, respectively. The pie charts at the nodes show the probabilities of gain/loss in ancestors. **b** Phylogenetic relationships of the five mirid bugs and associated copy numbers of LW1 opsins. The colors indicate the opsin types, and the numbers below represent the copy numbers of the opsins

**Fig. 3:**
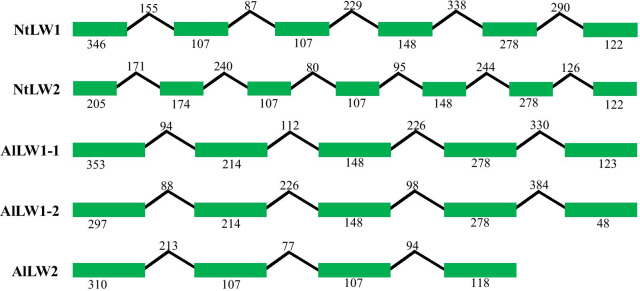
Genomic sequences of LW opsins in *Nesidiocoris tenuis* (NtLW) and *Apolygus lucorum* (AlLW). The rectangles represent exons, and the lines represent introns. The numbers represent the lengths in base pairs

Phylogenetic analyses based on translated amino acid sequences (Additional file 2) showed that all opsin genes formed four well-supported clades corresponding to four opsin types (Fig. 1). The LW opsins of mirid bugs were divided into two clades and showed a sister group relationship (Fig. 1), supporting a paralogous relationship between the two LW types in mirid bugs. LW opsin duplication likely occurred two times in *Ap. lucorum* and one time in the other four mirid bug species (Fig. 2b). In addition, the B opsins were likely lost in mirid bugs (Fig. 2a). Ancestral state reconstruction indicated that BG opsins are likely specific to *Drosophila*, which is classified within Diptera (Figs. 1, 2a). To determine the duplication mode of LW opsins in mirid bugs, we investigated the genomic structures of the LW opsins and found that all the LW opsins contained introns; however, different numbers of introns were observed in the *N. tenuis* (five introns in NtLW1 and six introns in NtLW2) and *Ap. lucorum* (four introns in AlLW1-1 and AlLW102 and three introns in AlLW2, but AlLW2 had an incomplete ORF) genes (Fig. 3, Additional file 3), excluding the possibility that duplication events occurred via retrogenes.

### Natural selection analyses

The CodeML branch model significantly rejected the neutral evolutionary hypothesis for the whole phylogeny of mirid opsins (likelihood ratio test [LRT] = 10,336.59, p < 0.001, Table 1). The multiple ratio model was more favored than the one-ratio model (LRT = 10,341.18, p < 0.001). The branch-specific ω values were 0.064, 0.059 and 0.050 for LW1, LW2 and other opsins, respectively. The ω value of LW1 was higher than that of LW2. Given this situation, we specifically tested whether natural selection acts on LW1 and/or LW2. However, both the CodeML (p > 0.05 for both LW1 and LW2) and BUSTED methods failed to detect a signature of positive selection, although the BUSTED analysis of LW1 provided a relatively small p value (0.099). Relaxation of selection pressures was also not detected with RELAX (p > 0.05). These results suggested that both LW1 and LW2 opsins of mirid bugs were under purifying selection.

**Table 1:** Selective patterns for LW opsins

Model	np^a^	Ln L^b^	Estimates of ω	Models compared	LRT^c^	*P* values
*Branch models*						
I: one ratio	118	− 44,881.38	ω = 0.053			
J: one ratio ω = 1	117	− 50,049.67	ω = 1	J vs. I	10,336.59	0
K: the LW1 lineage, LW2 lineage and the other branches have different *d*_*n*_*/d*_*s*_ ratios	120	− 44,879.08	ω_1_ = 0.064, ω_2_ = 0.059, ω_0_ = 0.050	I vs. K	10,341.18	0
M: each branch has its own ω	233	− 44,569.90	Variable ω by branch	I vs. M	622.96	0
*Branch-site models*						
N: LW1 lineage has ω = 1	120	− 44,755.26				
O: LW1 lineage	121	− 44,755.26		N vs. O	0	1
P: LW2 lineage has ω = 1	120	− 44,772.03				
Q: LW2 lineage	121	− 44,772.03		P vs. Q	0	1

^**a**^Number of parameters

^b^Natural logarithm of the likelihood value

^c^Twice the log-likelihood difference between the two models

### Expression analyses

**Fig. 4:**
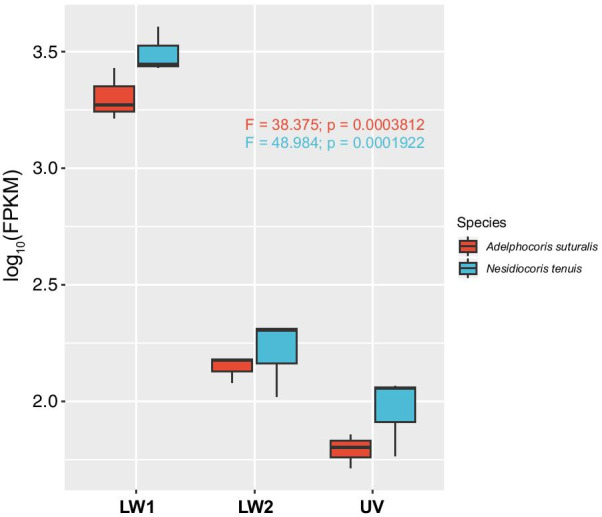
Expression levels of opsins in *Apolygus lucorum* and *Nesidiocoris tenuis*. The gene names are provided under the x-axis. The log-transformed FPKM values are displayed and were used for statistical analyses

The fragments per kilobase of exon per million fragments mapped (FPKM) method was used to determine the relative expression levels of the opsins in mirid bugs. LW1 opsins were significantly more highly expressed than LW2 and UV opsins in *N. tenuis* (df = 2, F = 48.984, P = 0.0001922) and *Ad. suturalis* (df = 2, F = 38.375, P = 0.0003812) (Fig. 4). However, there was no difference in expression levels between LW2 and UV opsins. The results were similar in *Ap. lucorum, Ad. fasciaticollis* and *Ad. lineolatus*, although there were no replicates (Additional file 4).

## Discussion

The evolution of color vision in animals, with opsins as a reference, has been well investigated in recent decades [25–27, 33, 36–38]. Through RNA-seq and phylogenetic analyses, we found that the LW and UV opsins of mirid bugs clustered with the opsins of other insects; however, no mirid opsins clustered with the B opsins, suggesting that B opsins have been lost in mirid bugs, similar to the situation in coleopteran insects [25–27]. Moreover, more than one contig clustered with the LW opsins of other insects, suggesting that LW opsin duplication occurred in mirid bugs (twice in *Ap. lucorum*).

Gene duplication is a common process in the evolutionary adaptation of organisms to the environment [39, 40]. There are two possible duplication modes, which are classified according to the sources of the duplicated genes: (1) DNA-based duplication, in which genomic DNA is used as a template and which can be detected according to the presence of regulated elements or introns [41–44]; and (2) RNA-based duplication, in which mRNA is used as a template (referred to as retrotransposition) and which can be detected on the basis of a lack of introns [45, 46]. Opsins, especially LW and UV opsins, have been duplicated in species from different insect orders, such as Coleoptera, Lepidoptera, Hymenoptera, Odonata and Orthoptera [23, 24, 26–31]. Both DNA- and RNA-derived duplications of LW opsins have occurred in lepidopteran insects [30, 31]. We did not detect duplication of UV opsins in mirid bugs, so we chose one phytozoophagous species (*Ap. lucorum*) and one zoophytophagous species (*N. tenuis*) to determine the duplication mode of LW opsins in mirid bugs. The results indicated that all of the LW opsins contained introns, suggesting that duplication events occurred with genomic DNA as a template. In lepidopteran insects, opsins usually contain seven introns that correspond to the seven transmembrane domains in the amino acid sequences of the opsins [30, 31]. However, the number of introns can differ within or between species of mirid bugs. For example, there are five introns in LW1 and six introns in LW2 in *N. tenuis*; in contrast, there are four introns in LW1-1/LW1-2 and three introns in LW2 in *Ap. lucorum*, although these genes have incomplete coding domains, suggesting that LW opsins in insects have different evolutionary modes. Herein, we used only adults to perform RNA-seq; thus, we may have missed some opsins with very low expression levels or stage-specific expression.

The evolutionary pattern of opsins in Arthropoda suggests that the functions of LW and UV opsins are fundamentally conserved [47]. Selection analyses have supported this possibility, indicating the occurrence of purifying or positive selection [24, 27, 29, 36, 48]. However, loss of B opsins has occurred in various insects, such as some species of Hemiptera and Coleoptera, suggesting that these opsins exhibit a different function and selection mode [25, 27]. Coleopterans include diurnal insects, negatively phototactic insects, and positively phototactic insects. These findings suggest that the loss of B opsins might not be related to the ambient light environment or to color vision. Interestingly, evidence regarding the physiology and evolution of opsin genes suggests that the function of B opsins is partially compensated for by LW and UV opsins in Coleoptera [25–27]. The LW genes of beetles have experienced strong purifying selection [26, 27], and strong purifying selection signals were detectable in the two LW opsins of the five investigated mirid bugs in our study, suggesting that these two genes possess a conserved function that is similar to those of the genes in other species. In noctuid moths, LW2 originated from retrotransposition and is under more relaxed purifying selection than LW1 [30]. However, LW2 in mirid bugs, which originated from DNA-based duplication events, is under selection pressure similar to that of LW1, suggesting that LW2 exhibits a more important biological function in mirid bugs than in noctuid moths.

Both variation and expression analyses have been used to investigate the evolution and functions of opsins [15, 49–52]. We further investigated the expression level of opsins in mirid bugs using the FPKM values obtained from RNA-seq. Retrogenes are typically presumed to be randomly inserted into the genome and to become pseudogenes due to the lack of a native promoter (except in cases in which a new promoter is acquired) [44, 46, 53]. However, duplicated genes based on DNA are expressed normally but at different levels based on the diversity of the regulatory apparatus (e.g., promoter) [54]. Interestingly, compared to UV opsins, LW2, which originated from retrotransposition, is expressed at very low levels (e.g., it is undetectable at the adult stage) in noctuid species [30]. However, both LW1 and LW2 are highly expressed in mirid bugs, although the expression levels of LW1 are significantly higher than those of LW2 and UV opsins, suggesting that LW2 plays a more important role in mirid bugs than in noctuid species. Adults of *Ap. lucorum* are significantly more attracted by green traps and green LEDs (515–518 nm) than by traps and LEDs of other colors, possibly because of the elevated expression levels of LW opsins in *Ap. lucorum* [10–12]. Moreover, mirid bugs usually show green-based body coloration, and the plants upon which they feed are also green, suggesting that the duplication of LW opsins might be related to mating choice and feeding in these insects. Further analysis of opsin evolution and expression levels could help us to develop novel management strategies exploiting the color vision and phototactic behavior of pests.

## Conclusions

We identified the opsins of five mirid bugs using RNA-seq. Phylogenetic analyses indicated the existence of UV opsins in all the mirid bugs as well as three LW opsins in *Ap. lucorum* and two LW opsins in the other four mirid bugs, suggesting that LW opsins were duplicated and that B opsins were lost during evolution. The duplicates of LW contained introns, implying that the duplication was DNA based. The strong purifying selection and relatively high expression levels suggested that the opsins in mirid bugs exhibit fundamental functions. Our results fill gaps in the body of knowledge regarding opsin evolution in insects.

## Methods

### Transcriptome analyses

*Ap. lucorum* adults were collected from a cotton field at the Langfang Experimental Station of the Chinese Academy of Agricultural Sciences (Hebei Province, China) in 2015 and were used for RNA-seq as described previously [55]. Briefly, total RNA was extracted from the whole bodies of fifty adults with TRIzol reagent (Invitrogen, Carlsbad, CA, USA), and mRNA was isolated using oligo(dT) magnetic beads. Then, the mRNA was broken into short fragments and used to synthesize cDNA with a SuperScript double-stranded cDNA synthesis kit (Invitrogen, Carlsbad, CA, USA). The short fragments (approximately 200 bp) were purified with a QIAquick PCR Purification Kit (Qiagen, Germany) and used to construct a cDNA library. Sequencing was performed via paired-end sequencing using an Illumina HiSeq™ instrument. De novo assembly was performed using Trinity (v2.0.6) [56]. Read mapping was performed with Bowtie 2 (https://sourceforge.net/projects/bowtie-bio/files/bowtie2). For functional annotation, the assembled contigs were aligned to the NR, STRING, SwissProt and KEGG databases with BLASTx (e-value ≤ 1E−5). For quantitative analyses, the read counts were calculated and then normalized to the FPKM values by using RSEM (v1.1.17) software [57, 58]. Statistical analyses of the gene expression levels were conducted using one-way ANOVA in R.

### Identification of opsin genes

Previously, we performed RNA-seq on *Ad. suturalis*, *Ad. fasciaticollis*, *Ad. lineolatus* and *N. tenuis* samples, and the data were submitted to the National Center for Biotechnology Information (NCBI), the SRA database (accession numbers: SRR6322944, SRR6322963, SRR6322964, SRR8259912, SRR8259810, SRR8259282, and SRR6322965) [35]. In the current study, we functionally annotated the visual opsins of these five mirid bugs. To understand the duplication modes of LW opsins in mirid bugs, we designed primers according to the reference sequences obtained from RNA-seq that amplified the opsin genes in template DNA from *N. tenuis* and *Ap. lucorum* in order to determine the genomic structures of the opsins (Additional file 1: Table S2). The PCR program was as follows: 30 s at 94 °C, 30 s at 55 °C, and 2 min at 72 °C for 40 cycles.

### Sequence alignment and phylogenetic/evolutionary analyses

We performed phylogenetic reconstruction, including additional opsins from other insect species. We used opsins from the genome of *Daphnia pulex* (the common water flea) as an outgroup. Fifty-seven opsin sequences were included in our analyses. Sequence alignment was performed using the codon model as implemented in PRANK [59]. Given the highly divergent patterns of these opsins, we used trimAl [60] to select blocks of conserved regions in the alignment for evolutionary inference. Phylogenetic analyses were performed using the maximum likelihood (ML) method in RAxML 7.3.2 [61] under the GTRGAMMA substitution model [62] for DNA and the PROTGAMMAJTTF model for proteins with 1000 and 100 replicates, respectively. Ancestral character states (gain or loss) of B and BG opsins and the associated uncertainty were estimated based on the phylogenetic relationships of insect orders using the ape package in R [63, 64].

### Selection assessment

We used the ML approach [65] to test differences in selection pressure between the two feeding habits using the CodeML program implemented in the PAML 4.5 package [66]. Specifically, we tested whether specific branch models and branch-site models could detect positive selection acting on particular lineages. Four hypotheses were evaluated: (1) that there is one *d*_*n*_*/d*_*s*_ ratio for all branches (one-ratio model; assumes that all branches have evolved at the same rate); (2) that the *d*_*n*_*/d*_*s*_ ratio = 1 for all branches (neutral model; neutral evolution for all branches); (3) that the LW1 lineage, the LW2 lineage and the other branches exhibit different *d*_*n*_*/d*_*s*_ ratios (ω_1,_ ω_2_ and ω_0_; three-ratio model; allows the foreground branch to evolve under a different rate); and (4) that each branch exhibits its own *d*_*n*_*/d*_*s*_ ratio. For the branch-site models, the LW1 and LW2 lineages were defined as foreground branches, and the remaining lineages were defined as background branches, as specified in the tree file using branch labels. The LRT was employed to determine whether the alternative model, indicating positive selection, was superior to the null model. The recently developed RELAX method [67], implemented in the program HYPHY [68], was employed to detect whether relaxation of selection pressure occurred at the LW1 and/or LW2 opsins of mirid bugs. In addition, we used the BUSTED method [69] to test whether a gene had experienced positive selection acting on at least one site among the LW1 and/or LW2 opsins of mirid bugs.

## Supplementary Information

**Additional file 1: Table S1.** Contigs showing high identity with opsins. Table S2: Primers used in this study.

**Additional file 2. **Sequences used for phylogenetic and selection analyses.

**Additional file 3. **Genomic sequences of LW opsins in *Nesidiocoris tenuis* and *Apolygus lucorum*

**Additional file 4. **Expression levels (FPKM values) of opsins in *Apolygus lucorum*, *Adelphocoris lineolatus* and *Adelphocoris fasciaticollis*.

## Data Availability

The datasets generated and analyzed during the current study are available in the Sequence Read Archive (SRA) repository under the accession numbers: SRR6371236 for *Apolygus lucorum*; SRR8259912, SRR8259810, and SRR8259282 for *Nesidiocoris tenuis*; SRR6322944, SRR6322963, and SRR6322964 for *Adelphocoris suturalis*; SRR6322965 for *Adelphocoris fasciaticollis*; and SRR6322463 for *Adelphocoris lineolatus*.

## Abbreviations

LW: Long-wavelength
UV: Ultraviolet
B: Blue
Gb: Gigabases
NCBI: National Center for Biotechnology Information
GEO: Gene expression omnibus
SRA: Sequence read archive
NtLW: LW opsin in *Nesidiocoris tenuis*
AlLW: LW opsin in *Apolygus lucorum*
FPKM: Fragments per kilobase of exon per million fragments mapped
LRT: Likelihood ratio test
ML: Maximum likelihood
nm: Nanometer
ORF: Open reading frame
