# Supplementary material for: The evolution of opsin genes in five species of mirid bugs: duplication of long-wavelength opsins and loss of blue-sensitive opsins
Source: BMC Ecol Evol. 2021 Apr 26;21:66. doi: 10.1186/s12862-021-01799-5 (PMC8074501; doi:10.1186/s12862-021-01799-5)
Supplement: Supplementary file 4 — Additional file 4. Expression levels (FPKM values) of opsins in Apolygus lucorum, Adelphocoris lineolatus and Adelphocoris fasciaticollis. [file 12862_2021_1799_MOESM4_ESM.docx]

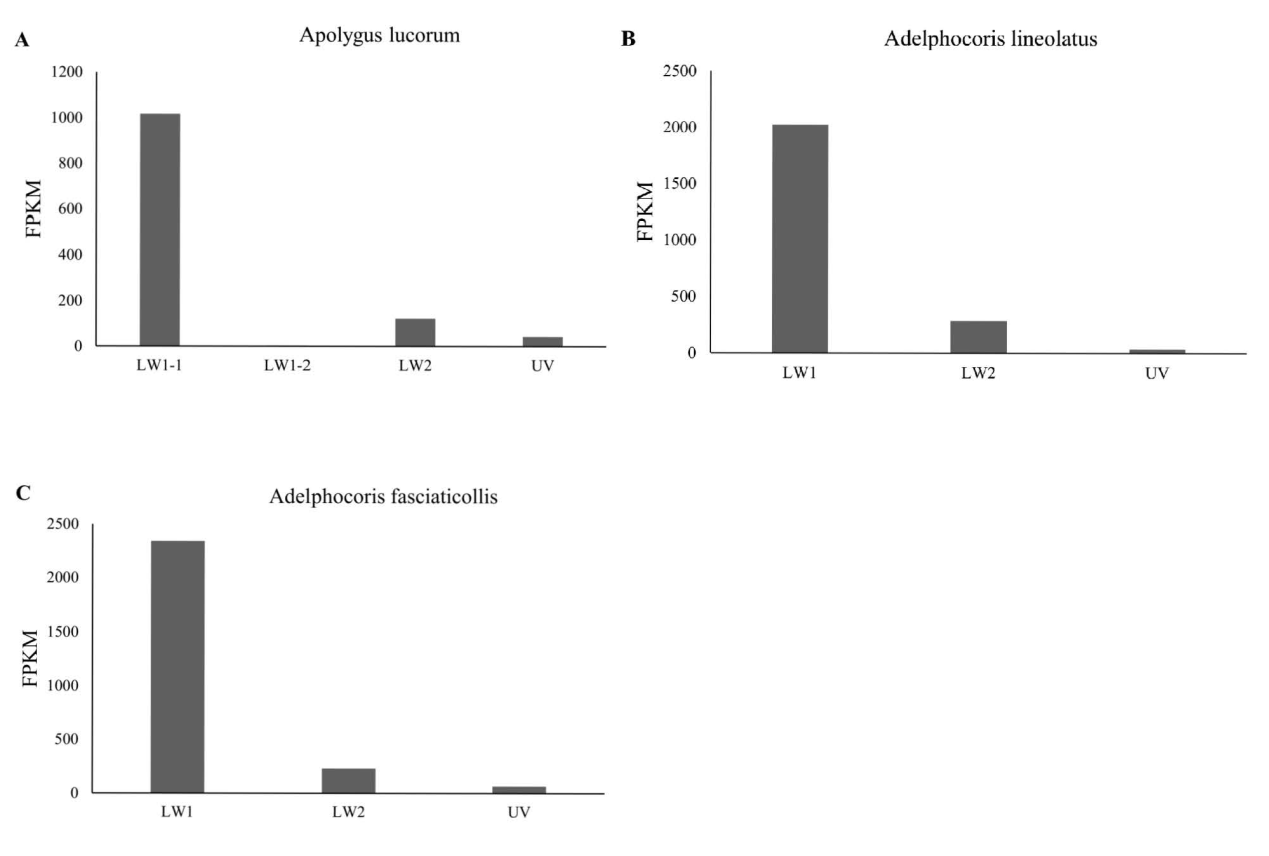


**Fig. S** Expression levels (FPKM values) of opsins in *Apolygus lucorum* (A), *Adelphocoris lineolatus* (B) and *Adelphocoris fasciaticollis* (C). The gene names were shown in Additional files 1-table S1.
